# Biotechnological development of plants for space agriculture

**DOI:** 10.1038/s41467-021-26238-3

**Published:** 2021-10-14

**Authors:** Yongming Liu, Gengxin Xie, Qichang Yang, Maozhi Ren

**Affiliations:** 1 Laboratory of Space Biology, Institute of Urban Agriculture, Chinese Academy of Agricultural Sciences, 610213 Chengdu, China; 2 Zhengzhou Research Base, State Key Laboratory of Cotton Biology, School of Agricultural Sciences of Zhengzhou University, 450000 Zhengzhou, China; 3 Hainan Yazhou Bay Seed Laboratory, 572025 Sanya, China; 4 Center of Space Exploration, Ministry of Education (Chongqing University), 400044 Chongqing, China

**Keywords:** Molecular engineering in plants, Molecular engineering in plants, Secondary metabolism

## Abstract

The ideal plant for cultivation in space would provide as many nutrients from as few inputs as possible. Here, we discuss how biotechnology could be used to produce a potato cultivar suitable for humans in space.

## Abstract (web summary)

The logistical and practical challenges of supplying food for long-term space missions are substantial. In this comment, the authors discuss potential biotechnological approaches that could be used to aid the production of food crops in space.

If humankind is ever to undertake long-term space missions and colonization, establishing an efficient space farming system would be essential for human survival in space. However, existing crops are not sufficiently cost effective and productive for use on space farms. Hence, we propose a Whole-Body Edible and Elite Plant (WBEEP) strategy for space crop improvement. Relying on plant biotechnology, the WBEEP strategy aims to develop crops with more edible parts, richer nutrient content, higher yields, and higher mineral nutrient use efficiencies for space farms.

Potato (*Solanum tuberosum* L.) is believed to be one of the top contenders for space agriculture due to the following advantages: (1) high harvest index and tuber yield and carbohydrate-rich tubers that can provide a large amount of energy for humans; (2) simple horticultural and food processing requirements; and (3) high tolerance against stresses with the ability to develop normally during spaceflight^1^. Importantly, potatoes can be asexually propagated through tubers and sexually propagated through seeds. Asexual reproduction can ensure the regeneration of food resources and stable nutritional value, while sexual reproduction can guarantee a higher propagation coefficient and lower storage and transportation costs^2^. However, potatoes cannot be efficiently cultivated in space until inherent defects related to their high solanine content, low yield and nutrient accumulation, and low fertilizer use efficiency are overcome. Below, we describe a WBEEP strategy for potato improvement that might create a WBEEP-potato for space farming (Fig. 1).

**Fig. 1:**
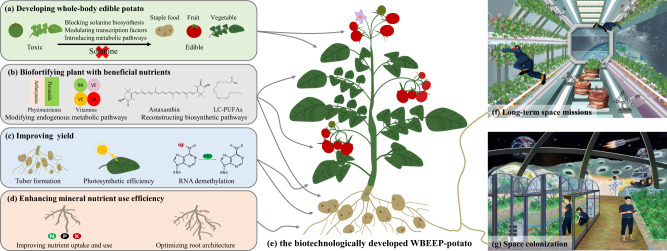
Biotechnological generation of WBEEP-potato and its applications. **a** Developing whole-plant edible potatoes. Blocking the biosynthesis of solanine, varying its transcriptional regulation, or introducing other metabolic pathways can reduce the accumulation of solanine and make potato stems, leaves and berries edible. **b** Biofortifying plants with beneficial nutrients. Biofortification can provide phytonutrients (e.g., flavonoids, anthocyanins, and carotenoids) by modifying endogenous metabolic pathways and astaxanthin or VLC-PUFAs (e.g., arachidonic acid) by reconstructing biosynthetic pathways. **c** Improving yield. Potato yield can be increased by improving tuberization, photosynthetic efficiency and RNA demethylation. **d** Enhancing mineral nutrient use efficiency. Nutrient utilization efficiency can be improved by modulating plant nutrient absorption, allocation and metabolism or optimizing root architecture. **e**–**g** The biotechnologically developed WBEEP-potato (**e**) is expected to be applied in long-term space missions (**f**) and space colonization (**g**).

## Developing whole-body edible plant for WBEEP-potato

Plants whose whole bodies are edible would be desirable for space farms because they can bring humans more food and reduce waste. However, potato stems, leaves, and berries are inedible. The aerial parts of potato plants contain accumulated solanine (primarily α-solanine and α-chaconine), which defends against pests and pathogens but is toxic to humans. In space farming systems, with highly controlled environments, solanine-mediated plant resistance would be unnecessary. If solanine were removed, the whole potato plant could potentially become edible. To block the accumulation of solanine in potato plants, biosynthesis can be targeted. For example, silencing or mutating genes encoding the cytochrome P450 enzyme GAME4, the dioxygenase DPS or the AP2/ERF transcription factor GAME9 greatly reduced solanine content^3–5^. Tomatoes can also produce toxic solanine (primarily α-tomatine) but can convert solanine into the nonbitter and nontoxic glycoside esculeoside A in fruits^6^. Since solanine metabolism involves several enzymatic reactions in common between potatoes and tomatoes, it might be possible to introduce solanine metabolism genes from tomatoes into potatoes to reduce solanine accumulation.

## Biofortification with beneficial nutrients for WBEEP-potato

Phytonutrients (e.g., flavonoids and anthocyanins) and vitamins are of great importance to human health. The body in space becomes more fragile and needs more nutrients^7^. However, several micronutrients in packaged foods are likely to break down under storage conditions in space, which makes it difficult for crews to obtain stable nutrients^8^. Therefore, it is desirable for people to obtain nutrition directly from fresh agricultural products. Considering the insufficient content of proteins, phytonutrients, vitamins, and other essential nutrients in potato tubers, it would be necessary to biofortify potatoes to fully meet the nutrient needs of the human body. Plants can be improved to synthesize vitamins and functional secondary metabolites by modifying endogenous metabolic pathways, including (1) increasing the precursor supply; (2) overexpression, relocation, or mutation of bottleneck enzymes; (3) silencing the undesired pathways; (4) blocking the competing pathways; (5) expansion of metabolic flow to reduce feedback inhibition; and (6) regulation of transcription factors. Through the application of the above strategies, potatoes that are rich in various vitamins, proteins, flavonoids, anthocyanins, and other nutrients have been developed^9^. Moreover, potato varieties containing canthaxanthin, astaxanthin, or very-long-chain polyunsaturated fatty acids (VLC-PUFAs) may be developed by reconstructing biosynthetic pathways^10^.

## Improving yield for WBEEP-potato

Tubers are the primary edible parts of potato plants. Potato tuberization is a complex biological process. Key regulators include photoreceptor phytochrome B (PHYB), transcription factor StCO, mobile signals (*StBEL5* and *POTH1* mRNA, StSP6A protein, and *miR172*), and sucrose transporters StSUT4 and StSP5G. Overexpression of *StSP6A*, *StPOTH1*, *StBEL5*, and *StmiR172* or inhibition of *StPHYB*, *StCO*, *StSUT4*, and *StSP5G* can be performed to improve tuberization^11^. Optimization of photosynthesis is one of the primary ways to increase crop yield, and potato tuberization also requires large amounts of photosynthetic products (especially sucrose) from the aboveground parts. Efforts are ongoing to increase photosynthetic efficiency by improving the carboxylation capacity of the Rubisco enzyme, enhancing the regenerative capacity of the carbon reduction cycle, optimizing the electron transport chain, and minimizing oxygenation and photorespiration^12^. Most of the abovementioned genetic engineering strategies for improving photosynthesis efficiency have been successfully applied in rice or tobacco and could hopefully be utilized to improve potato yield. For example, an artificially constructed photorespiratory bypass through the expression of a recombinant glycolate dehydrogenase polyprotein can significantly increase the photosynthetic efficiency and tuber yield by reducing photorespiration and improving CO_2_ uptake^13^. Recently, modulating plant RNA m^6^A methylation has become an efficient way to improve plant growth and crop yield. Transgenic expression of the human RNA demethylase FTO to reduce m^6^A levels in potatoes led to ~50% increases in tuber yield and aerial biomass^14^.

## Enhancing mineral nutrient use efficiency for WBEEP-potato

Crop growth and development require many mineral elements, including nitrogen, phosphorus, and potassium. The cost of transporting fertilizers from Earth is very high. Therefore, it is necessary to improve crop nutrient utilization efficiency to reduce fertilizer consumption. Genetic modification could be carried out to enhance plant nutrient absorption, allocation, and metabolism or to optimize root architecture^12^. Nitrogen is one of the most important elements required by plants. Glutamate dehydrogenases (GDHs) from lower organisms show a higher affinity for NH_4_^+^ and a stronger ammonia assimilation ability. Heterologous expression of GDHs that have higher affinity for NH_4_^+^ than plant GDHs can improve the nitrogen use efficiency of many crops, including potatoes, and ensure that crops can obtain high yields under low-nitrogen conditions^15^. Phosphorus is another essential element for plant growth, and phosphite fertilizers can promote an increase in the yield and quality of potato tubers. Expression of phosphite dehydrogenase (*ptxD*) from *Pseudomonas* spp. allows rice and cotton to metabolize phosphite in addition to phosphate^16^, and its role in potato is worth exploring. Potatoes need more potassium fertilizer than nitrogen or phosphate fertilizers for growth and quality. Heterologous expression of the Arabidopsis K^+^ channel *AKT1* and its activators *CBL1*, *CBL9*, and *CIPK23* can increase the efficiency of potassium uptake from the soil in several crops, and overexpressing the K^+^ transporter *HAK5* may increase the efficiency of potassium uptake in many crops under potassium-limited conditions^17^. Recently, genetically manipulating the root system architecture has become an emerging strategy to increase nutrient acquisition and yield in tuber crops, but genes that can be exploited in potatoes must be mined.

## Future prospects for WBEEPs

Experiments have been conducted to show how plants grow and develop in space, but clear space agriculture remains in its infancy. Only green leafy vegetables such as lettuce and mustard are currently grown for food on the International Space Station^18^. Thus, to bring more plants to space farms, we propose the WBEEP approach for crop improvement. A comprehensively applied WBEEP could provide sufficient and nutritious food for humans in space with minimal fertilizer consumption. As more anti-nutritional factor biosynthesis mechanisms are revealed, and strategies for improving nutrition, yield, and fertilizer use efficiency are developed, the WBEEP approach could be implemented on more crops. While the practical cultivation of WBEEPs in space for food might not be achievable any time soon, we suggest that considering the incremental advances needed to achieve such a goal might be beneficial not only for space agriculture but also for conventional agriculture.
